# Focussed ultrasound thalamotomy and functional MRI as windows to neural networks underlying essential tremor

**DOI:** 10.1016/j.neurot.2026.e00893

**Published:** 2026-03-28

**Authors:** Stephen Tisch

**Affiliations:** a School of Clinical Medicine, University of New South Wales, Sydney, New South Wales, Australia; b Department of Neurology, St Vincent's Hospital Sydney, St Vincent's Health Network, New South Wales, Australia

**Keywords:** Essential tremor, MRgFUS, Resting state fMRI

A question frequently asked by essential tremor (ET) patients is “why do I shake” often followed up by “how do we fix it”. The two questions are closely related, and the answer to each can shed light on the other. MRI guided focussed ultrasound (MRgFUS) ablation of the thalamus ventral intermediate (VIM) nucleus is now an accepted and widely performed effective treatment for medication resistant ET [1,2]. Clinical benefit after MRgFUS thalamotomy is both immediate and sustained [3,4] with most ET patients receiving the treatment unilaterally, and an emerging role for staged bilateral treatment [5]. MRgFUS has revived the use of thalamotomy as a means of neuromodulation and presents an opportunity to study a new generation of patients with modern methods such as MRI, to better understand networks underlying disease states and effective treatment. Some MRI studies have attempted to correlate location of the lesion with clinical outcome and demonstrated that proximity and overlap with dentatorubrothalamic tract [6] and putative thalamic “sweet spots” [7] may predict clinical benefit. Some studies have utilised advanced MRI techniques to study brain networks before and after MRgFUS thalamotomy in ET. Findings have included increased functional connectivity between the left thalamus and dorsal premotor cortex [8], increased activity in the sensorimotor cortex with decreased activity in the posterior cingulate cortex [9], enhanced corticostriatal connectivity [10] and increased connectivity in sensorimotor, cerebellar and visual networks [11] after MRgFUS thalamotomy for ET. Overall, results have not been entirely consistent between studies and the salient “tremor network” in ET mediating improvement is not yet universally agreed.

In this issue of Neurotherapeutics, Liu and colleagues use graph theory and connectomic network reconstruction to analyse structural and resting state fMRI datasets in a series of 37 ET patients before and 6 months after MRgFUS VIM thalamotomy [12]. Clinical outcome data were collected using standardised rating scales. Graph theory is computationally demanding and models brain regions as nodes and edges, which provides a measure of local nodal specialization and connectedness [13]. Existing studies utilising graph theory analysis in ET have shown abnormal network properties [14], and the authors hypothesized that changes might be observed after clinically effective VIM ablation. In terms of clinical benefit, significant tremor reduction was achieved in this cohort, in line with world-wide experience. The MRI analysis demonstrated significant increase in local specialization and connectedness referred to in graph analysis as an increase in *small-worldness* and *normalized clustering coefficient*. This change to the network is thought to reflect improved efficiency through smaller but locally highly connected nodes. Other measures including *characteristic path length, normalized characteristic path length, global efficiency, and local efficiency* were not altered after MRgFUS. There were also trends for increased connectivity between frontoparietal and subcortical (thalamic) modules, and frontoparietal to frontotemporal-parietal modules. These network changes were associated with increased *betweenness centrality* between the left superior frontal gyrus, right superior parietal lobule, and left postcentral gyrus. There were no significant correlations between the MRI changes and the clinical scores. The pattern of changes observed at baseline and after MRgFUS partially align with existing fMRI studies in ET before and after thalamotomy. These findings, together with the present study by Liu et al. [12], have shifted our concept of pathophysiological networks in ET beyond the cerebello-thalamo-cortical circuit to an extended tremor network of multiple cortical regions including attentional, salience, agency, error correction and visual areas [14,15]. It is important to appreciate the limitations of the present study by Liu et al. [12], and other studies of this type, including sample size, lack of healthy control groups and methodological limitations, and inherent assumptions that are made in resting state (rs) fMRI analysis [13]. Moreover, the rs fMRI changes after MRgFUS are meaningful at a group level but are not yet useful at an individual patient level to guide treatment planning or predict outcome. Nevertheless, the scientific opportunity to elucidate brain networks underlying disease states and treatment effect, provided by a new generation of patients undergoing MRgFUS lesional therapies for movement disorders and other conditions, is an important and steadily growing area of study. Understanding the networks underlying movement disorders like ET is an essential step in improving mechanistic understanding of disease and treatment outcomes, as we move from the older concept of targeted brain therapies simply disrupting anatomical nodes, to the current concept of network-wide neuromodulation. Focussed ultrasound ablative interventions sit squarely within neuromodulatory approaches [16], as the effects on brain networks extend far beyond the lesion itself, as the present study shows. ET joins a growing list of movement disorders considered network disorders or “circuitopathies” including dystonia and Parkinson's disease. The answer for the ET patient who asks ‘why do I shake” has become more complicated but is also gradually becoming better understood. As to whether ET can be “fixed”, MRgFUS VIM ablation, whilst not a cure, has provided a highly effective non-invasive option for improving tremor.

## Author contributions

Professor Stephen Tisch was the sole author and contributor to the invited commentary titled “Focussed ultrasound thalamotomy and functional MRI as windows to neural networks underlying Essential Tremor” commenting on the accepted article by Liu et al. “Functional Network Reorganization Following VIM-MRgFUS for Essential Tremor” forthcoming in Neurotherapeutics, article ID: NEUROT-D-25-00768R1.

## Declaration of competing interest

The authors declare the following financial interests/personal relationships which may be considered as potential competing interests: Stephen Tisch reports was provided by St Vincent's Hospital Sydney. If there are other authors, they declare that they have no known competing financial interests or personal relationships that could have appeared to influence the work reported in this paper.
